# Effects of scheduled ibuprofen combined with diclofenac suppository on postoperative pain in children undergoing pectus excavatum correction: a randomized controlled trial

**DOI:** 10.3389/fphar.2026.1901666

**Published:** 2026-10-28

**Authors:** Yan Jiang, Lihong Chen, Jia Li, Junli Dong

**Affiliations:** 1 Department of Anesthesiology, Shanghai Children’s Hospital, Shanghai JiaoTong University, Shanghai, China; 2 Department of Thoracic Surgery, Shanghai Children’s Hospital, Shanghai JiaoTong University, Shanghai, China

**Keywords:** diclofenac sodium, ibuprofen, Nuss procedure, pectus excavatum, postoperative analgesia

## Abstract

**Background:**

Non-steroidal anti-inflammatory drugs (NSAIDs) are widely used for postoperative acute pain management. However, the safety and efficacy of postoperative scheduled oral ibuprofen remain unclear in children. The purpose of this study was to evaluate the efficacy and safety of scheduled ibuprofen combined with diclofenac suppository for postoperative analgesia in children following the Nuss procedure for pectus excavatum.

**Methods:**

Sixty patients who underwent the Nuss procedure from September 2024 to August 2025 were randomly divided and assigned to either the experimental or control group. The experimental group received scheduled ibuprofen combined with a diclofenac suppository, whereas the control group received a diclofenac suppository and ibuprofen on demand. The postoperative visual analog scale (VAS) scores, supplemental opioid consumption, time to first ambulation, length of hospital stay, and incidence of adverse events were recorded for both groups.

**Results:**

The VAS scores at 6–24 h postoperatively were significantly lower for the experimental group than the control group (*p* < 0.05). The experimental group required less opioids than the control group (62.4 ± 5.7 vs. 67.5 ± 6.4 µg, *p* = 0.002; difference: −5.167 µg, 95% confidence interval: −8.325 to −2.008). Additionally, the time to first ambulation (5.9 ± 0.6 vs. 6.4 ± 0.7 h, *p* = 0.005; difference: −0.486 h, 95% confidence interval: −0.924 to −0.076) and length of hospital stay (6.8 ± 0.8 vs. 7.3 ± 0.8 days, *p* = 0.022; difference: −0.500 days, 95% confidence interval: −0.924 to −0.076) were shorter for the experimental group than the control group. The incidence of adverse events was similar for both groups (*p* > 0.05).

**Conclusion:**

This study indicates that scheduled ibuprofen combined with diclofenac suppository provides effective analgesia following the Nuss procedure, reduces opioid consumption, and promotes recovery. No serious NSAID-related adverse events were observed during the short postoperative observation period. However, the study was not powered to establish safety or exclude clinically important differences in uncommon adverse events.

**Clinical Trial Registration:**

https://www.chictr.org.cn/, identifier ChiCTR2400089927.

## 1 Introduction

Pectus excavatum is the most common congenital chest wall deformity, for which the Nuss procedure (minimally invasive repair of pectus excavatum) has become the gold standard of treatment owing to advantages such as less trauma, faster recovery, and better cosmetic outcomes (David, 2022; Hebra et al., 2016). However, this procedure involves the introduction of a curved stainless-steel bar behind the sternum to correct the depressed chest wall, which increases tension on the chest wall tissues, muscles, and bones to cause dramatic postoperative pain (Lo et al., 2020). In such cases, inadequate analgesia can adversely affect the patient outcomes (Fang et al., 2026). Therefore, postoperative acute pain management is receiving increased clinical attention in recent times.

Postoperative pain from the Nuss procedure impairs respiratory functions, causing atelectasis, pulmonary infections, and other complications, while limiting early ambulation and hindering recovery (Breglio et al., 2023). Although traditional opioid-based analgesic regimens are effective, they often cause nausea, vomiting, constipation, respiratory depression, and urinary retention, with a notably higher incidence of such adverse effects in pediatric patients (Chiu et al., 2024). The current therapeutic trend favors a “low-opioid, opioid-sparing” regimen involving local anesthetics combined with acetaminophen and/or non-steroidal anti-inflammatory drugs (NSAIDs), following the Procedure-Specific Postoperative Pain Management (PROSPECT) recommendations (Die et al., 2021).

Ibuprofen and diclofenac sodium are both NSAIDs that exert anti-inflammatory and analgesic effects by inhibiting cyclooxygenase (COX) activity and reducing prostaglandin synthesis. Recently, NSAIDs are being increasingly used in multimodal analgesia because of their advantages, such as non-central action, non-addictive properties, and relatively fewer adverse reactions (Chou et al., 2016). The present study aimed to investigate the efficacy and safety of scheduled ibuprofen combined with diclofenac sodium for postoperative analgesia in children who underwent the Nuss procedure.

## 2 Materials and methods

This study was conducted in accordance with the guidelines of the Declaration of Helsinki and approved by the Ethical Committee of Shanghai Children’s Hospital (approval number: 2024R093-F02, chairman: Prof. Zb. Lv). This study was registered with the Chinese clinical trial registration (number: ChiCTR2400089927, date: 19 September 2024). Written informed consent for participation was obtained from the guardians of the children.

From September 2024 to August 2025, we enrolled 60 patients with pectus excavatum into the trial. The inclusion criteria were as follows: (1) diagnosed with pectus excavatum and scheduled for the Nuss procedure; (2) age 9–17 years; (3) physical status I–II according to the American Society of Anesthesiologists (ASA); (4) normal lung function or preoperative pulse oxygen saturation ≥96%. The exclusion criteria were as follows: (1) history of peptic ulcer; (2) coagulation disorders; (3) history of asthma; (4) allergies to opioids or NSAIDs (ibuprofen/diclofenac sodium); (5) liver or kidney dysfunction. The children were randomly divided into the experimental and control groups (30 cases each) using a random number table.

### 2.1 Randomization

Randomization was performed by opening an opaque envelope containing a sequential number before inducing anesthesia. These numbers were generated preoperatively by a nurse using an online randomization tool. The investigators, anesthetists, surgeons, and children were blinded to the group allocation. The pain management team handled postoperative pain treatment according to group allocation and was not involved in this study.

### 2.2 Anesthesia protocol

Upon arrival in the operating room, the patients were routinely monitored with electrocardiography, pulse oximetry, and non-invasive blood pressure monitoring. After establishing venous access, anesthesia was induced with atropine (0.01 mg/kg), midazolam (0.05 mg/kg), propofol (2.5 mg/kg), sufentanil (0.4 μg/kg), and cisatracurium (0.15 mg/kg). An appropriately sized endotracheal tube was then inserted for intubation, followed by mechanical ventilation. The ventilation parameters included a tidal volume of 6–8 mL/kg, a respiratory frequency of 12–14 breaths/min, and a positive end-expiratory pressure of 3 cm H_2_O with adjustments to maintain the end-tidal CO_2_ between 30 and 45 mmHg. Anesthesia was maintained with propofol at 6 mg/kg/h combined with 1%–3% sevoflurane to maintain the systolic blood pressure within 20% of the baseline value. Intermittent boluses of cisatracurium (0.05 mg/kg) and sufentanil (0.1 μg/kg) were administered. When the mean arterial pressure (MAP) decreased to below 65 mmHg or 80% of the baseline, ephedrine (0.1–0.2 mg/kg) was administered. All surgical procedures were standard Nuss operations performed by the same team of cardiothoracic surgeons. At the end of the surgery, anesthetics were discontinued and intravenous ondansetron was administered at 0.75 mg/kg to prevent postoperative nausea and vomiting (PONV). Additionally, a wound infiltration nerve block was performed using 10 mL of 0.3% ropivacaine. A patient-controlled intravenous analgesia (PCIA) pump was connected, which contained sufentanil at 2 μg/kg diluted to 100 mL, with an infusion rate of 2 mL/h, a lockout interval of 15 min, and a bolus dose of 2 mL. The patients were transferred to the cardiac care unit (CCU), and tracheal extubation was performed when the patients had a spontaneous tidal volume of ≥6 mL/kg and were awake.

### 2.3 Interventions

To the children in the experimental group, diclofenac sodium suppository of 50 mg was administered rectally at the end of surgery, followed by oral ibuprofen (Shanghai Johnson & Johnson Pharmaceutical Co., Ltd., China) at a dose of 7.5 mg/kg every 8 h.

To the children in the control group, diclofenac sodium suppository of 50 mg was administered rectally at the end of surgery, and ibuprofen was administered orally at a dose of 7.5 mg/kg as needed (VAS score ≥4).

Based on rectal diclofenac administration, oral ibuprofen was given by the anesthesia nurses (pain management team) every 8 h or as needed depending on the group. An assistant anesthetist also instructed the patients on how to use the analgesia pump and assessed the postoperative pain intensity. The pain assessments were performed every hour after extubation. A bolus dose was administered when the VAS pain score was ≥4, and nalbuphine 0.2 mg/kg was given if this score remained ≥4.

### 2.4 Measurements

1. Pain scores: The VAS (range: 0–10) was used to assess postoperative pain intensity at cough.
2. Analgesic consumption: The total consumption of opioid (sufentanil) via PCIA within 24 h postoperatively and number of boluses were recorded.
3. Recovery parameters: The time to first ambulation and length of hospital stay were recorded.
4. Adverse reactions: PONV, dizziness, constipation, gastrointestinal bleeding, and liver/kidney function injuries were recorded over a 3-day postoperative period. Gastrointestinal bleeding was defined as a positive fecal occult blood test. Liver function injury was defined as a serum alanine aminotransferase (ALT) concentration of > 80 U/L, while kidney function injury was defined as a serum creatinine concentration of ≥156 μmol/L within 48 h.

### 2.5 Sample size calculation

The primary outcome was the postoperative pain score at 6 h postoperatively. Based on a pilot study (10 cases per group) that showed 6-h postoperative pain scores of 3.2 ± 0.5 and 3.7 ± 0.6, an estimated minimum of 25 cases was required for each group with an α of 0.05 and power of 0.9. Hence, the sample size of each group was increased to 30 to account for an anticipated 20% dropout rate.

### 2.6 Statistical analysis

The data were analyzed using SPSS 27.0. Continuous data with a normal distribution were expressed as mean ± standard deviation (x̄ ± s) and compared using independent sample t-tests. Continuous data with a non-normal distribution were expressed using the median value and compared using the Mann–Whitney test. Categorical data were expressed as numbers (percentages) and compared using the chi-squared or Fisher test. A *p*-value of < 0.05 was considered statistically significant.

## 3 Results

A total of 62 children were enrolled in the study initially and 60 children completed the trial ultimately (Figure 1). There were no statistically significant differences between the two groups in terms of age, height, weight, body mass index (BMI), gender, Haller index, nalbuphine use, or durations of surgery and anesthesia (Table 1).

**FIGURE 1:**
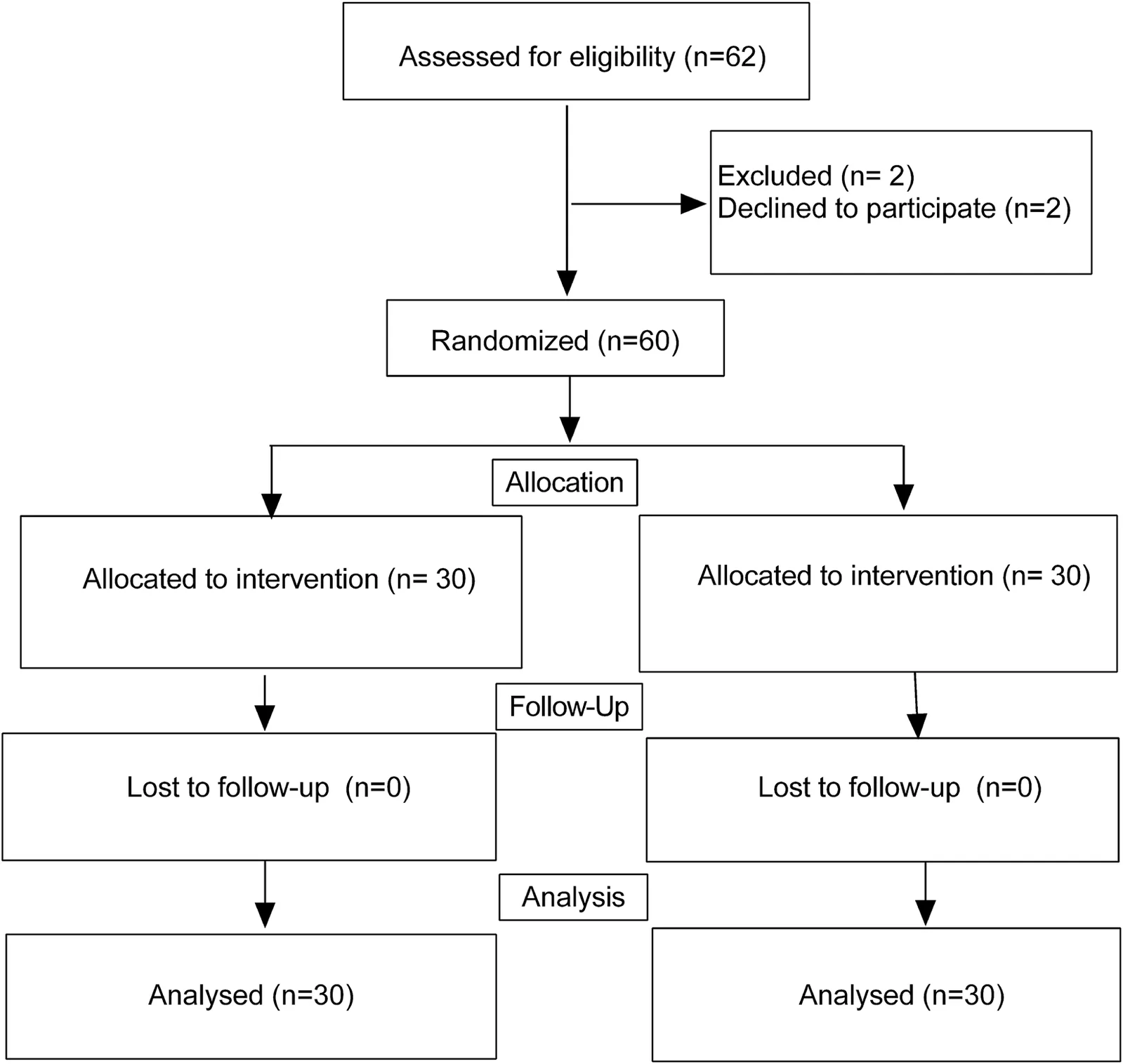
Flow diagram of our study.

**TABLE 1:** Data of the children enrolled in the study (n = 30).

Index (unit)	Experimental group	Control group	*p*-value
Age (year)	13.7 ± 1.5	14.6 ± 1.7	0.872
Weight (kg)	51.6 ± 7.0	52.4 ± 6.2	0.666
Height (cm)	164.2 ± 9.0	167.1 ± 7.1	0.170
Body mass index (kg/m^2^)	18.9 ± 1.8	18.5 ± 1.1	0.286
Gender (boy/girl)	25/5	26/4	0.999
Haller index	2.9 ± 0.4	2.8 ± 0.3	0.283
Duration of anesthesia (min)	92.5 ± 16.6	87.7 ± 10.5	0.179
Duration of surgery (min)	61.3 ± 13.8	57.4 ± 10.7	0.221
Consumption of sufentanil (µg)	62.4 ± 5.7	67.5 ± 6.4	0.002*
Number of boluses	5.4 ± 1.5	7.1 ± 1.9	<0.001*
Nalbuphine use (n)	3	7	0.166
Time to first ambulation (h)	5.9 ± 0.6	6.4 ± 0.7	0.005*
Length of hospital stay (d)	6.8 ± 0.8	7.3 ± 0.8	0.022*

Data are expressed as mean ± standard deviation or number.

* indicates p < 0.05.

### 3.1 Pain scores

The experimental group had significantly lower VAS scores at 6, 12, and 24 h postoperatively compared to the control group (*p* < 0.05). However, there was no significant difference in the VAS scores between the two groups at 48 h postoperatively (*p* > 0.05; Figure 2).

**FIGURE 2:**
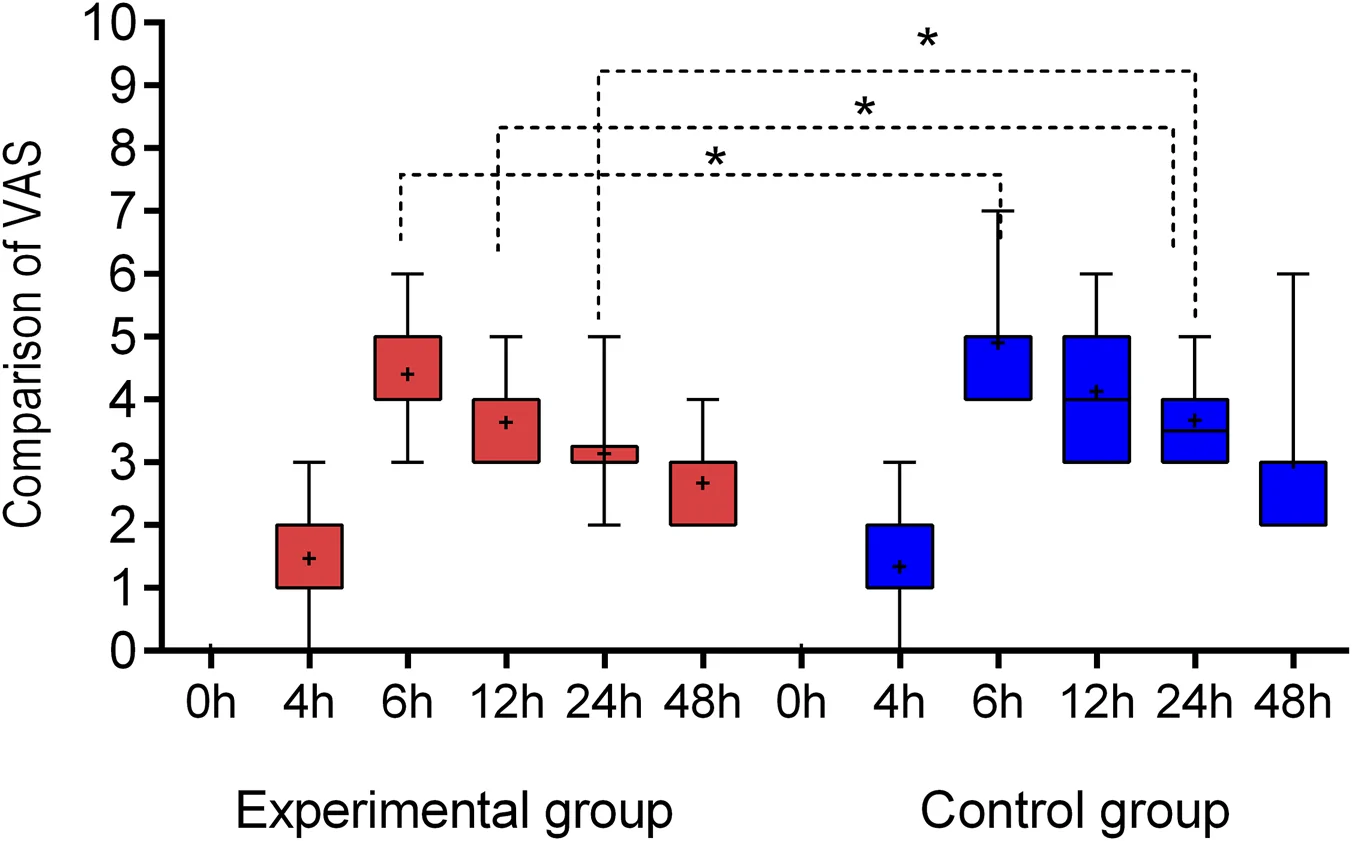
Comparison of postoperative visual analog scale (VAS) scores between the two groups with *p < 0.05.

### 3.2 Analgesic consumption and recovery

The experimental group showed significantly lower sufentanil consumption within 24 h postoperatively (62.4 ± 5.7 vs. 67.5 ± 6.4 μg, *p* = 0.002; difference: −5.167 μg, 95% confidence interval (CI): −8.325 to −2.008) and fewer boluses (5.4 ± 1.5 vs. 7.1 ± 1.9, *p* < 0.001; difference: −1.833, 95% CI: −2.702 to −0.965) compared to the control group. The time to first ambulation (5.9 ± 0.6 vs. 6.4 ± 0.7 h, *p* = 0.005; difference: −0.486 h, 95% CI: −0.924 to −0.076) and postoperative length of hospital stay (6.8 ± 0.8 vs. 7.3 ± 0.8 days, *p* = 0.022; difference: −0.500 days, 95% CI: −0.924 to −0.076) were shorter for the experimental group than the control group (see Table 1).

### 3.3 Adverse events

The incidence of PONV was lower in the experimental group; however, there was no significant difference between the two groups (*p* > 0.05). No significant instances of gastrointestinal bleeding, coagulation disorders, or renal injury were observed in either group (Table 2).

**TABLE 2:** Adverse events in the children enrolled in the study (out of n = 30).

Index	Experimental group	Control group	*p*-value
Liver function injury (n)	0	0	—
Kidney function injury (n)	0	0	—
Respiratory depression (n)	0	1	0.999
Dizziness (n)	2	3	0.999
Constipation (n)	0	0	—
Nausea and vomiting (n)	2	4	0.671
Gastrointestinal bleeding (n)	0	0	0.999

Data are expressed as numbers (percentage).

## 4 Discussion

This study shows that scheduled ibuprofen combined with a diclofenac suppository provides effective analgesia, reduces opioid consumption, and shortens the time to first ambulation and length of hospital stay following the Nuss procedure in children.

The quality of pain management following the Nuss procedure for pectus excavatum significantly influences patient recovery (Breglio et al., 2023). Although the Nuss procedure is minimally invasive, the pain resulting from the bar-induced traction on the ribs, sternum, and muscles is often severe and is primarily inflammatory and somatic in nature (Kelly et al., 2019). Although the traditional opioid-centric analgesia model is effective, it is associated with adverse effects such as nausea, vomiting, and ileus, which significantly hinder feeding and mobilization. In adolescents, severe nausea and vomiting may even lead to complications such as pneumothorax or bar displacement (Chiu et al., 2024). Consequently, multimodal analgesia has become the preferred approach in postoperative pain management; this strategy involves combining analgesics with different action mechanisms to achieve synergistic effects, reduce the dosage of individual drugs, and minimize adverse reactions (Cone et al., 2023).

The results of our study demonstrate that adding ibuprofen to routine wound infiltration along with opioid analgesia significantly reduces the VAS scores in the 6–24 h interval postoperatively. This suggests that ibuprofen inhibits both peripheral and central prostaglandin synthesis, thereby alleviating the inflammatory responses and pain sensitization induced by surgical trauma effectively. The experimental group also exhibited significantly lower opioid consumption, confirming the beneficial “opioid-sparing” effect of ibuprofen. Moreover, reducing opioid use has been shown to have significant clinical benefits in decreasing opioid-related complications (Wick et al., 2017). This is particularly critical for patients with pectus excavatum because vomiting increases the intrathoracic pressure, causing severe incisional pain and potentially compromising the stability of the bar. Additionally, the experimental group ambulated approximately 6 h on average and experienced a shorter hospital stay by approximately 0.5 days, which align closely with the principles of enhanced recovery after surgery (ERAS) (Wharton et al., 2020; Cramm et al., 2021). Effective pain control provides the necessary conditions for early ambulation, thereby promoting recovery and reducing the length of hospital stay.

When using ibuprofen, attention should be paid to its potential risks. In this study, no gastrointestinal bleeding or renal function injuries occurred in both groups. This outcome is attributed to several factors: (1) short observation period (postoperative 3 days); (2) exclusion of patients with a history of peptic ulcers or renal insufficiency; (3) use of a diclofenac suppository to reduce the dose of ibuprofen required. Although some studies have suggested that NSAIDs may impair bone healing in surgeries involving bones (such as the sternum or ribs), this risk is negligible in the Nuss procedure because the bar serves a stabilizing role and does not involve bone healing (Pountos et al., 2012).

## 5 Conclusion

This study indicates that scheduled ibuprofen combined with a diclofenac suppository could provide effective analgesia following the Nuss procedure while reducing opioid consumption and promoting recovery. No serious NSAID-related adverse events were observed during the short postoperative observation period. However, this study was not powered to establish safety or exclude clinically important differences in uncommon adverse events.

## Data Availability

The raw data supporting the conclusions of this article will be made available by the authors without undue reservation.
